# Senior Adults: Does Body Image Matter?

**DOI:** 10.1093/geroni/igaa057.3262

**Published:** 2020-12-16

**Authors:** Keri Larsen, Myia Graves, Ashley Bowers, Valerie Saba, Lauren Himel

**Affiliations:** 1 Southeastern Louisiana University, Hammond, Louisiana, United States; 2 Southeastern Louisiana University, Loranger, Louisiana, United States; 3 Southeastern Louisiana University, Prairieville, Louisiana, United States

## Abstract

Through the theoretical framework of the Social Comparison Theory, the current study will examine general attitudes and perceptions of body image in senior adults who are currently participating in organized recreational activities. Participants between the ages of 50 years of age and older participating in organized recreational programs in the Southeast will be administered the Sociocultural Attitudes Toward Appearance Scale (SATAQ) to measure participants’ body image as influenced by general media, athletic and sport figures, as well as pressure to conform to the media ideal. The Figure Rating Scale will be administered, and is composed of nine drawings of bodies that progressively increase in size from very thin to overweight. Pearson product moment coefficient of correlation will be used to determine the association of scores between the SATAQ and Figure Rating Scale.

